# The Outcome of the Oxidations of Unusual Enediamide Motifs Is Governed by the Stabilities of the Intermediate Iminium Ions

**DOI:** 10.1371/journal.pone.0047224

**Published:** 2012-10-19

**Authors:** Muneer Ahamed, Bun Chan, Paul Jensen, Matthew H. Todd

**Affiliations:** 1 School of Chemistry, The University of Sydney, Sydney, New South Wales, Australia; 2 ARC Centre of Excellence for Free Radical Chemistry and Biotechnology, Victoria, Australia; California Institute of Technology, United States of America

## Abstract

We compare the results from the oxidation of two unusual “enediamide” motifs (3,4-dihydropyrazin-2(1*H*)-ones), where a double bond is flanked by two amides. In one case the oxidation led to a ring-opened product arising from the cleavage of the double bond, and in the other a rare *cis*-dioxygenated compound was obtained. Both products have been characterized by X-ray crystallography. The outcomes of the key reactions are rationalized based on calculated free energies of intermediates.

## Introduction

**Figure 1:**
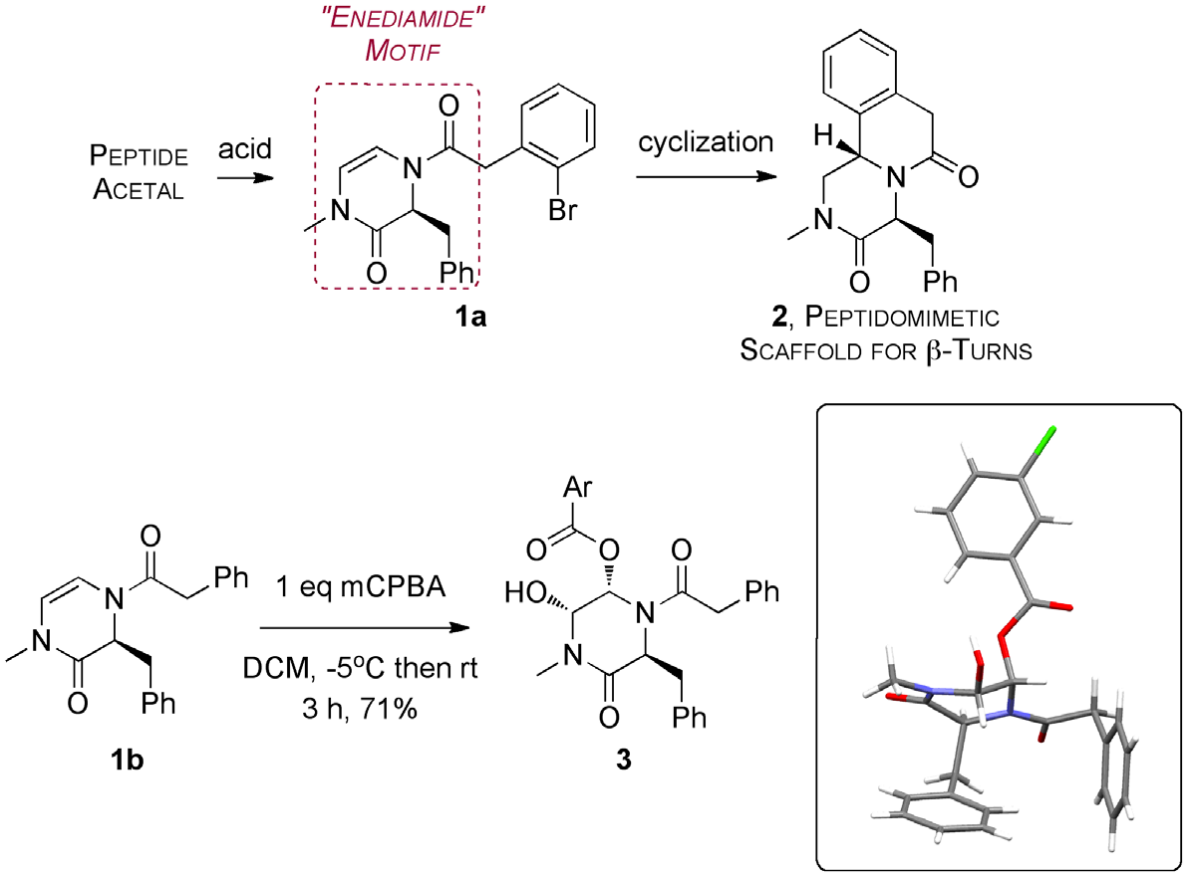
Previous work on the synthesis of peptidomimetic scaffolds, and the unusual stereochemical outcome of the epoxidation of enediamide 1b reported here (Ar = 3-chlorophenyl) proven with X-ray crystallography (inset).

During the course of previous work on the synthesis of peptidomimetic scaffolds (**2**, Figure 1) we synthesized an unusual enamide-based heterocycle consisting of a double bond linking two amides (**1a**). [1] One of the striking features of the chemistry was the degree of stereocontrol of ring closure (to give **2**) by the aromatic ring attached to the exocyclic amide, caused by the pseudoaxial orientation of the other benzyl group (derived from the amino acid phenylalanine) blocking one face of the double bond.

During further exploration of this chemistry we obtained an unusual result, now reported here. Attempted epoxidation of **1b** gave not the expected epoxide but a derivative **3**, which was identified by X-ray crystallography (inset, Figure 1). The benzoic acid derived from the reagent attached itself to the initial oxidation product of the reaction, but the relative stereochemistry of the new substituents was *cis.* This contrasts with the much more typical *trans*- ring opening in such reactions. [2]–[3] but which has been seen in cases where diastereoselectivity in the opening of the epoxide is influenced by adjacent stereocentres [4] (including quaternary stereocentres generated during the epoxidation [5]–[7]) or chiral auxiliaries.[8]–[11] In the present case the epoxide presumably opened to give an intermediate acyliminium ion that was attacked from the opposite face of the heterocyclic ring because of the steric effect of the pendant benzyl group.

**Figure 2:**
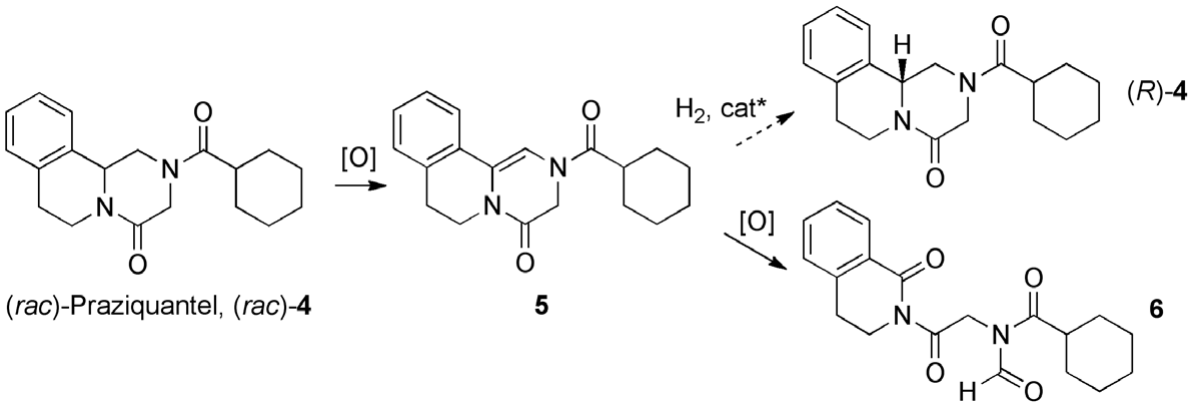
Synthesis of enediamide 5, an intermediate in the proposed enantioselective synthesis of the drug praziquantel (4), and precursor to a ring-opened oxidation product 6.

**Figure 3:**
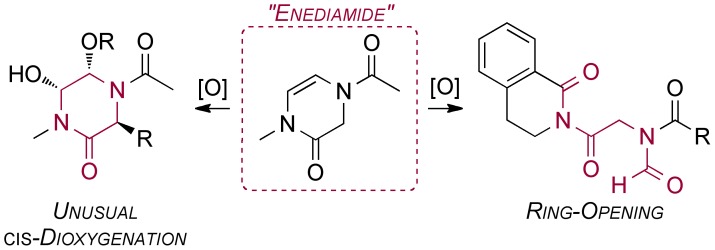
Comparison of enediamide outcomes.

The motif of interest in **1** may be referred to as an “enediamide” (formally a 3,4-dihydropyrazin-2(1*H*)-one, or (in non-IUPAC approved nomenclature) a Δ^5^-2-oxopiperazine); the structure contains two amide nitrogen lone pairs able to act as π-donors to the double bond. Enediamides have been only occasionally reported in the literature.[1], [12]–[16] Their oxidation [16]–[18] has not been well explored. Most relevant was a report of enediamide **5** (Figure 2). [19] Oxidation of this structure by *m*CPBA was reported to give a ring-opened product (**6**) derived from cleavage of the double bond, rather than formation of an epoxide or dihydroxylated derivative. The identity of this compound was suggested on the basis of NMR studies. This contrasting behaviour of enediamide **5** has recently also become of interest to us. Our laboratory has been leading an open source project on the internet to find an enantioselective synthesis of the important drug praziquantel (PZQ, **4**). [20] One of the approaches suggested on the project website [21] was the oxidation of PZQ to give the enediamide **5**, which could be subjected to an asymmetric hydrogenation to generate the desired (*R*)-PZQ. The preparation of **5** is facile, [19], [22] but during the course of screening oxidants, we frequently obtained low yields and multiple byproducts. Given the apparent difference in outcomes of the oxidations of enediamides **1b** and **5** (Figure 3) we sought to confirm the identity of the product arising from **5** and address why this reaction outcome was so different to that with enediamide **1b**. We presumed (as did Davies *et al.* in a study of epoxidation of an enamide *vs*. its ring opening [9]) that the outcome is governed by stability of the intermediate iminium ions, but wished to provide evidence for this presumption.

## Results and Discussion

**Figure 4:**
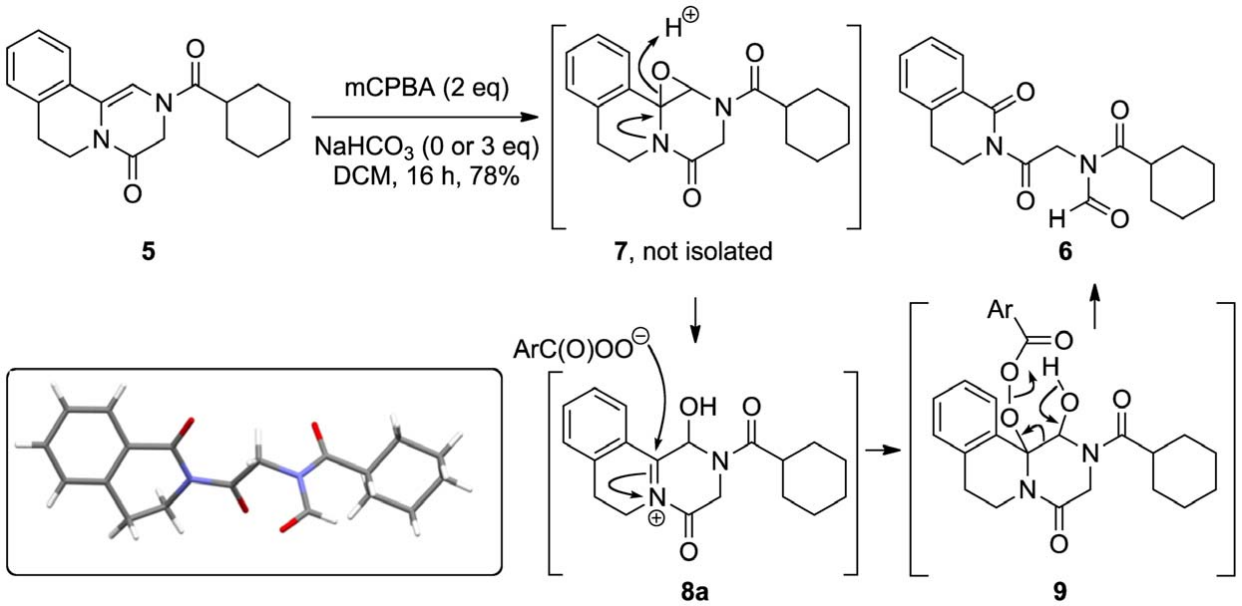
Attempted epoxidation of enediamide 5 gives ring opened product 6, characterized by X-ray crystallography (inset).

In our hands, treatment of enediamide **5** with one equivalent of *m*CPBA gave a yield of 40% of the ring-opened product, with the remaining mass balance being starting material. Since the production of **6** requires two equivalents of oxidant, this yield is formally 80%. Use of two equivalents of *m*CPBA gave the ring-opened product in 78% yield. The use of NaHCO_3_ as base did not affect these yields. Crystals of **6** suitable for X-ray diffraction were obtained by slow evaporation from a mixed solvent system of Et_2_O and MeOH, unambiguously confirming the double-imide identity of this molecule (Figure 4).

A plausible mechanism for the formation of this compound involves formation of the epoxide **7** as a first step. This epoxide is unstable with respect to protonation and opening *via* formation of an acyliminium ion (*e.g.*, **8a**, an isomeric compound **8b** is considered below) which would be attacked by *m*CPBA to give **9**, triggering ring opening *via* a Grob fragmentation. [9], [11] The formation of *cis*-diols from solvolysis of cyclohexene oxides was shown to be promoted by electron-rich aromatic groups at the 1-position, with the reaction occurring *via* a transition state with significant carbocationic character. [23].

Given that the PZQ-enediamide **5** may itself be formed by an oxidation of PZQ, the same ring opened product **6** can also be obtained directly from PZQ (**4**). The original report of this reaction gave a reaction time of one week with the use of two equivalents of *m*CPBA. We treated PZQ with a range of equivalents of the oxidant (Table 1). With three equivalents of *m*CPBA conversion of starting material was still incomplete after 16 hours. A trace amount (observed in all cases when the reaction was analysed by TLC) of **5** was formed in all cases, but 63% of the ring opened product **6** was generated with the rest of the mass balance being unreacted PZQ. Five equivalents of oxidant were necessary to consume all the starting material and give the ring opened product in high yield. The relative yields suggest enediamide **5** reacts more quickly with *m*CPBA than the starting material **4**.

**Figure 5:**
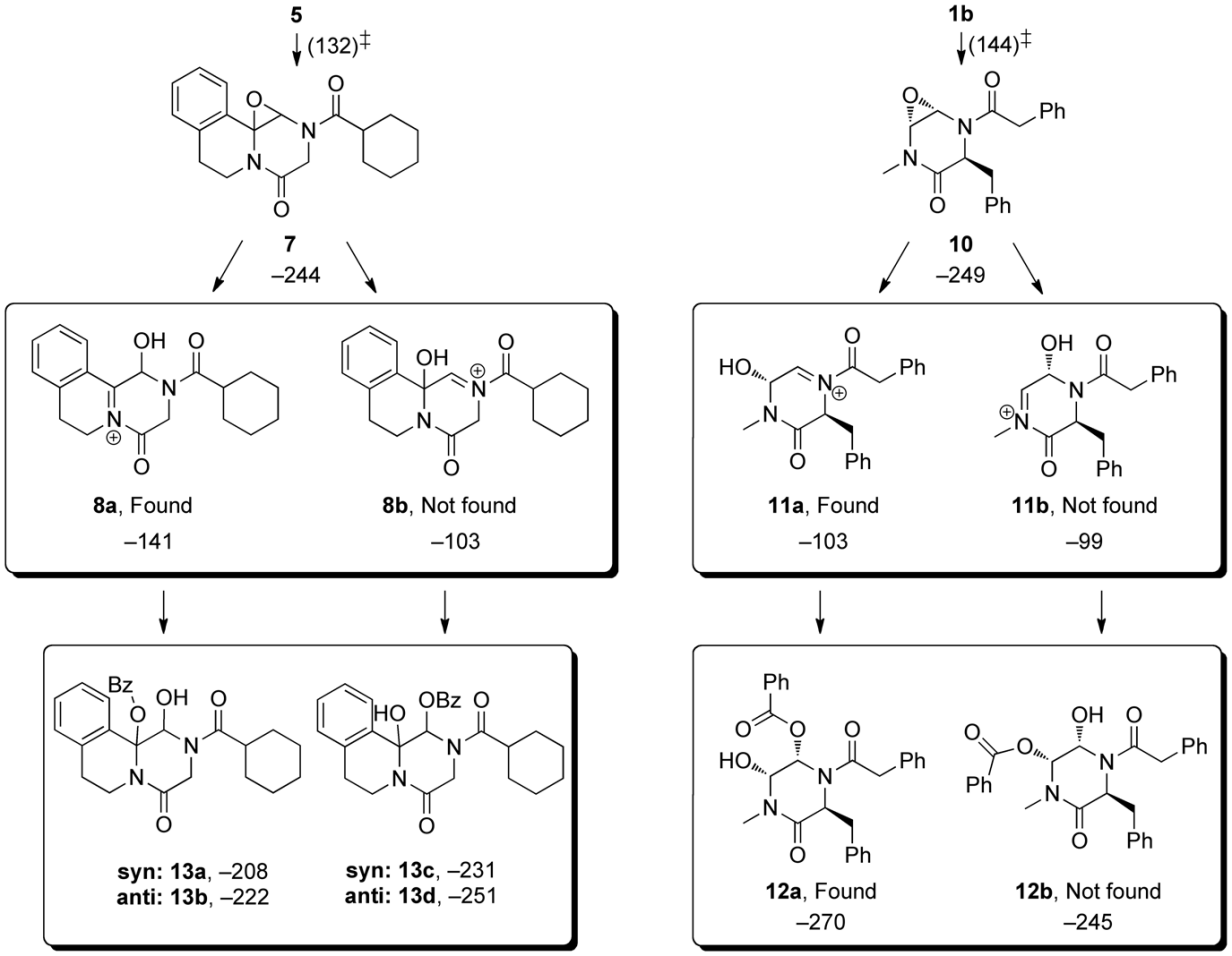
Regioselectivity in the opening of enediamide epoxides, and a comparison of observed selectivity with calculated M06-2X/6-311+G(3df,2p) free energies (298 K, kJ mol^–1^) of iminium ions and possible products.

**Figure 6:**
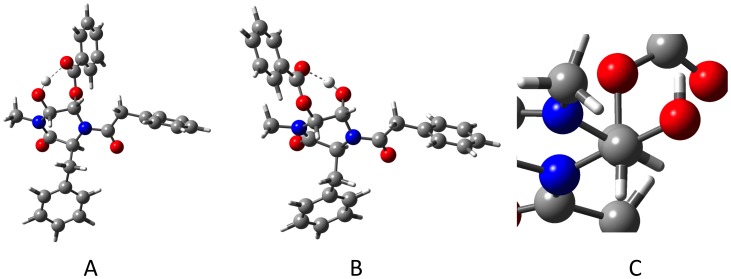
B3-LYP/6-31G(d) minimum energy conformations of A) 12a (found) and B) 12b (not found, showing eclipsed ester and *N*-Me groups) and C) view along the C2–C3 bond showing gauche arrangements of oxygen and nitrogen substituents.

In the case of the formation of **3**, only one regioisomer was formed, according to the uncomplicated ^1^H NMR spectrum that gave rise to the crystalline sample used for structure determination. If the attachment of the benzoate is reversible, then the more stable ring opened product has been obtained. The same result would be obtained were the 3-chlorobenzoyl group to migrate between the 2- and 3-oxy substituents of the product. Modeling of the two possible iminium ions (**11a** and **11b**, Figure 5) and the regioisomeric products (**12a** and **12b**) suggests that the iminium ion **11a** is preferred to a small extent, and that the product **12a** is preferred to a large extent. Assuming the transition state barriers broadly reflect these values, it would appear that thermodynamic arguments explain the outcome (rather than necessitating a Curtin-Hammett calculation of transition state energies). The large difference in product free energies for **12a** and **12b** are steric in origin. The exocyclic phenethylacyl group forces the adjacent oxo substituent into an axial position. The adjacent (*cis*) oxo substituent (to which there is a hydrogen bond) is therefore equatorial, and inevitably clashes with the *N*-methyl group (Figure 6, A and B). There is a “double gauche effect” in the product observed in which the electronegative oxygen and nitrogen substituents both generate this stereoelectronic stabilization (Figure 6, C). [7] The corresponding values for the iminium ion for the ring opening reaction (**8a** and **8b**) show the expected intermediate is heavily favored where the iminium ion double bond is conjugated with the aromatic ring, but the same product would ultimately be formed irrespective of which of **8a** or **8b** forms. However, the fact that **8a** is of considerably lower energy than **8b** is significant in that were attack by benzoic acid to occur we would expect products **13a** or **13b** to form (rather than **13c** or **13d**). The higher energies of these benzoate-trapped compounds *vs*. the epoxide **7** imply their formation is not favourable.

What of the different outcomes of the reactions? In the case of enediamide **5**, oxidative ring opening is observed, but this is not seen for enediamide **1b**. The pathways from starting materials to iminium ions involve a transition state that is slightly lower in energy for **5**, but it is illuminating to instead consider the pathways available to the iminium ion. In both cases there is a very large free energy gain in forming the epoxide from the iminium ion, for which the process is essentially barrierless. Alternatively, the trapping of the iminium ion by benzoate yields the stable adducts **13** (from **8**) and **12** (from **11**) and these trapping reactions are also quite exothermic. In the case of **11a** such adduct formation is more favourable than the formation of the epoxide, while for **8a**, formation of epoxide is more likely. Presumably the rapid formation of **12** from **11** means the benzoate counterion arising from the oxidation event attaches to the molecule before it can diffuse away – and this may be another reason for the defined stereochemical outcome. In contrast the ready formation of the neutral molecule **7** from **8a** allows for the diffusion away of the benzoate and permits the reaction of **8a** with mCPBA, triggering the ring opening reaction. Thus **8a** (formed preferentially to **8b**) coexists with residual oxidant because its trapping with benzoate is less favorable than for **11**.

### Conclusions

The difference in outcome of the epoxidation of two enediamides arises from the relative free energies of epoxides or benzoate-trapped products that can be formed from the intermediate iminium ions. These differences permit one intermediate iminium ion to react with residual oxidant and give a ring-opened product.

The lack of oxidative ring opening of enediamides such as **1b** and the defined stereocontrol of the oxidation reaction make these scaffolds attractive for the future stereoselective synthesis of polycyclic peptidomimetic motifs, were intramolecular nucleophiles, such as pendant alcohols, to be employed along with the oxidant.

**Table 1:** Direct oxidation of *rac*-PZQ (**4**) to ring opened product **6**.

Eq *m*CPBA	Yields (%)	
	Reclaimed PZQ (4)	6
1	60	39 (117)^a^
2	53	44 (66)
3	23	63 (63)
5	trace	91 (91)

a This slight over-production of **6** was also noted in the original report,^13^ where two equivalents of *m*CPBA gave 81% of the ring opened compound, representing a yield of 122% based on oxidant. Presumably there is a small degree of adventitious oxidation arising from atmospheric oxygen.

Yields in parentheses are yields based on moles of oxidant. Reaction conditions: DCM, rt, 16 h.

## Materials and Methods

### A) Synthesis

A general description of synthetic methods may be found in Text S1.

#### (2*S*,3*R*,6*S*)-6-Benzyl-3-hydroxy-4-methyl-5-oxo-1-(2-phenylacetyl)piperazin-2-yl 3-chlorobenzoate (3)

To (*S*)-3-benzyl-1-methyl-4-(2-phenylacetyl)-3,4-dihydropyrazin-2(1*H*)-one (**1b**, 30 mg, 0.094 mmol) [1] in DCM (3 mL) at −5°C was added *m*CPBA (16 mg, 1 eq, pre-washed in aqueous buffer pH 7.5 and dried). The reaction mixture was allowed to warm to rt over 1 h, stirred at rt for 2 h and concentrated *in vacuo*. The residue was purified by flash column chromatography (EtOAc-petroleum ether, 1∶4, ramping to EtOAc) to give the *hemiaminal* as white needles (22 mg, 71%). ^1^H NMR (400 MHz, CDCl_3_): δ 1.70 (s, 1H, OH), 2.71 (s, 3H, NMe), 3.07 (br d, 1H, *J ca*. 15, ArC*H*HCH), 3.22 (br s, 1H, CHO), 3.51 (dd, 1H, *J* 17.0 & 3.0, ArCH*H*CH), 3.58 (d, 1H, *J* 7.6, CHO), 3.79 (d, 1H, 14.8), 3.97 (d, 1H, *J* 14.4) (ABq, ArCH_2_CO), 4.80–4.87 (m, 1H, NCHCO), 6.63–6.76 (m, 2H), 6.84 (s, 1H), 7.03 (t, 2H, *J* 7.2), 7.14 (t, 1H, *J* 6.8), 7.27–7.48 (m, 5H), 7.55 (d, 1H, *J* 8.0), 7.80 (d, 1H, *J* 8.0), 7.89 (s, 1H) (Figure S1). IR (evaporated DCM solution) 3281 br, 1727, 1673, 1639 cm^−1^. MS (FAB) *m/z*: 493 (65%, MH^+^). HRMS (FAB) Calcd. for C_27_H_26_ClN_2_O_5_ (MH^+^): 493.15248, found 493.152250.

CCDC 833830 contains the supplementary crystallographic data for this compound. These data can be obtained free of charge from The Cambridge Crystallographic Data Centre *via* www.ccdc.cam.ac.uk/data_request/cif. The cif file is provided (Cif S1).

#### 2-(Cyclohexanecarbonyl)-6,7-dihydro-2H-pyrazino[2,1-a]isoquinolin-4(3H)-one (5)

2-(Cyclohexanecarbonyl)-3,6,7,11b-tetrahydro-1H-pyrazino[2,1-a]isoquinolin-4-one (*rac*-**4**, 0.500 g, 1.602 mmol) was mixed with sulfur (0.102 g, 3.204 mmol, 2 eq), under N_2_ and the mixture was melted with a hot air gun and kept at *ca.* 180°C for 15 min. The resultant dark brown oil was purified by flash chromatography (hexane:ethyl acetate, 5∶1) to afford the *enediamide* as a pale yellow solid (0.315 g, 63%). m. p. 131–134°C (lit. [19] 128–132°C). NMR data complicated by presence of minor rotamer: ^1^H NMR (300 MHz, CDCl_3_): δ 1.26–1.88 (m, 10H, cy), 2.63–2.71 (m, 1H, cy), 2.93 (t, 2H, *J* 5.7, H^7^), 3.93 (t, 2H, *J* 5.7, H^6^), 4.45 (s, 2H, H^3^), 6.78 (s, 1H, H^1^), 7.19–7.57 (m, 4H, Ar) (Figure S2). ^13^C NMR (75 MHz, CDCl_3_): δ 26.1 (m, 2 major peaks), 29.2, 29.4, 38.8, 41.4, 45.9, 48.7, 106.0, 123.1, 127.7, 127.7, 128.4, 128.8, 134.5, 164.3, 174.5 (C-11b not visible) (Figure S3). IR (evaporated CHCl_3_ solution): 2952, 2870, 1652, 1463 cm^−1^. MS (ESI) *m/z*: 311.1 [(MH)^+^, 100%], 333.3 [(MNa)^+^, 55%]. HRMS (ESI) Calcd. for C_19_H_22_N_2_NaO_2_ (MNa^+^): 333.15735. Found: 333.15765. Spectroscopic data matched those in the literature. [19].

#### *N*-Formyl-*N*-(2-oxo-2-(1-oxo-3,4-dihydroisoquinoline-2(1yl)ethyl)cyclohexanecarboxamide (6)

2-(Cyclohexanecarbonyl)-6,7-dihydro-2H-pyrazino[2,1-a]isoquinolin-4(3H)-one (**5**, 0.100 g, 0.322 mmol, 1.0 eq) in DCM (5 mL) was stirred with *m*CPBA (0.056 g, 0.322 mmol, 1.0 eq). Stirring was continued at rt for 16 h. The reaction mixture was diluted with DCM (15 mL) and washed with H_2_O (2×5 mL), the organic portion was dried (MgSO_4_) and concentrated *in vacuo*. The crude material was purified by flash column chromatography (EtOAc:hexane 1∶4) to afford the *ring opened compound* as a white solid (44 mg, 40%).

m. p. 117.0–118.5°C (lit. [19] 125°C). ^1^H NMR (500 MHz, CDCl_3_): δ 1.25–1.96 (m, 10H, cy), 2.75-2-83 (m, 1H, cy), 3.02 (t, 2H, *J* 6.0, H^4^), 4.10 (t, 2H, *J* 6.0, H^3^), 5.14 (s, 2H, H^9^), 7.26 (d, 1H, *J* obscured by solvent peak, Ar), 7.40 (t, 1H, *J* 7.6, Ar), 7.53 (d, 1H, *J* 7.5, Ar), 8.14 (d, 1H, *J* 7.7, Ar), 9.32 (s, 1H, H^10^) (Figure S4). ^13^C NMR (500 MHz, CDCl_3_): δ 25.6, 25.7, 28.0, 29.5, 42.3, 42.8, 46.6, 127.5, 127.6, 128.7, 129.7, 133.9, 140.2, 162.2, 165.9, 170.4, 177.0 (Figure S5). IR (evaporated CHCl_3_ solution): 2930, 2857, 1690, 1305, 747 cm^−1^. MS (ESI) *m/z*: 365.3 [(MNa)^+^, 100%]. HRMS (ESI) Calcd. for C_19_H_22_N_2_NaO_4_ (MNa^+^): 365.14718 Found: 365.14743. Spectroscopic data matched those in the literature. [19].

CCDC 816518 contains the supplementary crystallographic data for this compound. These data can be obtained free of charge from The Cambridge Crystallographic Data Centre *via* www.ccdc.cam.ac.uk/data_request/cif. The cif file is provided (Cif S2).

### B) Computational Methods

Standard density functional theory calculations were carried out with the GAUSSIAN 09 [24] program. Geometries were obtained at the B3-LYP [25] level of theory with the 6-31G(d) basis set. The vibrational frequencies of stationary points were inspected to ensure that they corresponded to minima on the potential energy surface. Improved relative energies were obtained at the M06-2X [26] level with the 6-311+G(3df,2p) basis set. Zero-point vibrational energies, thermal corrections to enthalpy and entropies were obtained using B3-LYP/6-31G(d) harmonic vibrational frequencies, scaled using appropriate literature scale factors. [27] We account for the effect of solvation using the SMD [28] continuum model with CH_2_Cl_2_ parameters. Energies in the text correspond to relative free energies at 298 K. Optimised structure coordinates and electronic energies may be found in Table S1.

## Supporting Information

Figure S1 ^1^H NMR (300 MHz) spectrum of (2*S*,3*R*,6*S*)-6-benzyl-3-hydroxy-4-methyl-5-oxo-1-(2-phenylacetyl)piperazin-2-yl 3-chlorobenzoate (**3**).
(TIFF)

Figure S2 ^1^H NMR spectrum of 2-(cyclohexanecarbonyl)-6,7-dihydro-2H-pyrazino[2,1-a]isoquinolin-4(3H)-one, (**5**).
(TIFF)

Figure S3 ^13^C NMR spectrum of 2-(cyclohexanecarbonyl)-6,7-dihydro-2H-pyrazino[2,1-a]isoquinolin-4(3H)-one, (**5**).
(TIFF)

Figure S4 ^1^H NMR Spectrum of *N*-formyl-*N*-(2-oxo-2-(1-oxo-3,4-dihydroisoquinoline-2(1yl)ethyl)cyclohexanecarboxamide, (**6**).
(TIFF)

Figure S5 ^13^C NMR Spectrum of *N*-formyl-*N*-(2-oxo-2-(1-oxo-3,4-dihydroisoquinoline-2(1yl)ethyl)cyclohexanecarboxamide, (**6**).
(TIFF)

Table S1 B3-LYP/6-31G(d)-optimized structures and M06-2X/6-311+G(3df,2p) electronic energies for various species.
(DOC)

Text S1 General Experimental Information.
(DOC)

Cif S1 Crystallographic data file for compound **3.**
(CIF)

Cif S2 Crystallographic data file for compound **6.**
(CIF)
