# Supplementary figures and images for: The Outcome of the Oxidations of Unusual Enediamide Motifs Is Governed by the Stabilities of the Intermediate Iminium Ions
Source: PLoS One. 2012 Oct 19;7(10):e47224. doi: 10.1371/journal.pone.0047224 (PMC3477162; doi:10.1371/journal.pone.0047224)

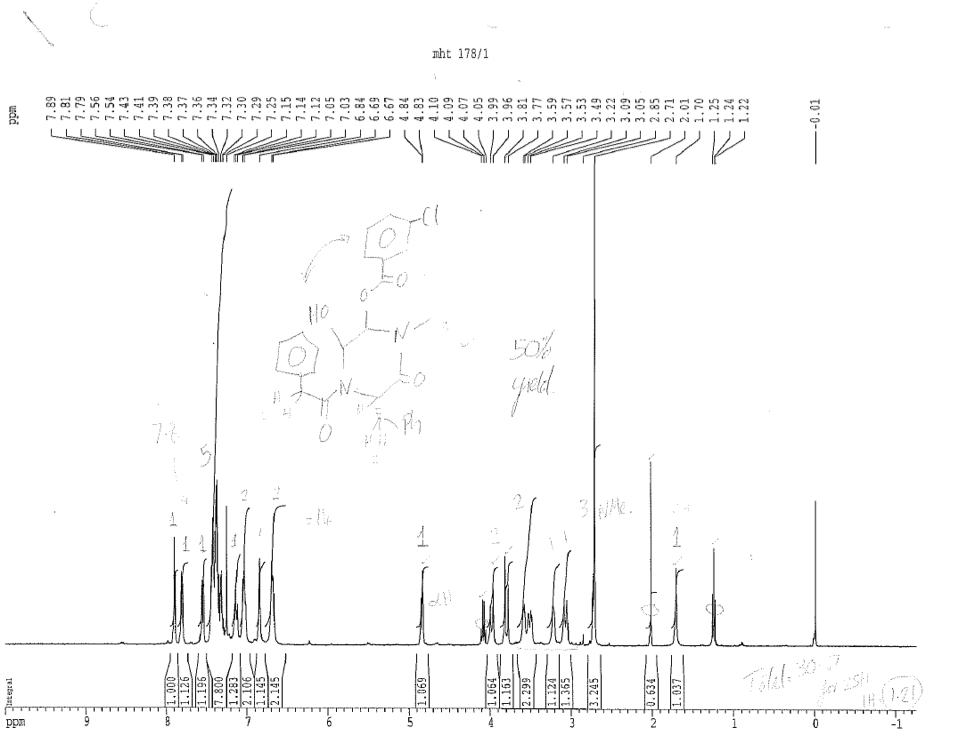

Supplement: Figure S1 — 1H NMR (300 MHz) spectrum of (2S,3R,6S)-6-benzyl-3-hydroxy-4-methyl-5-oxo-1-(2-phenylacetyl)piperazin-2-yl 3-chlorobenzoate (3). (TIFF) [file pone.0047224.s001.tif]

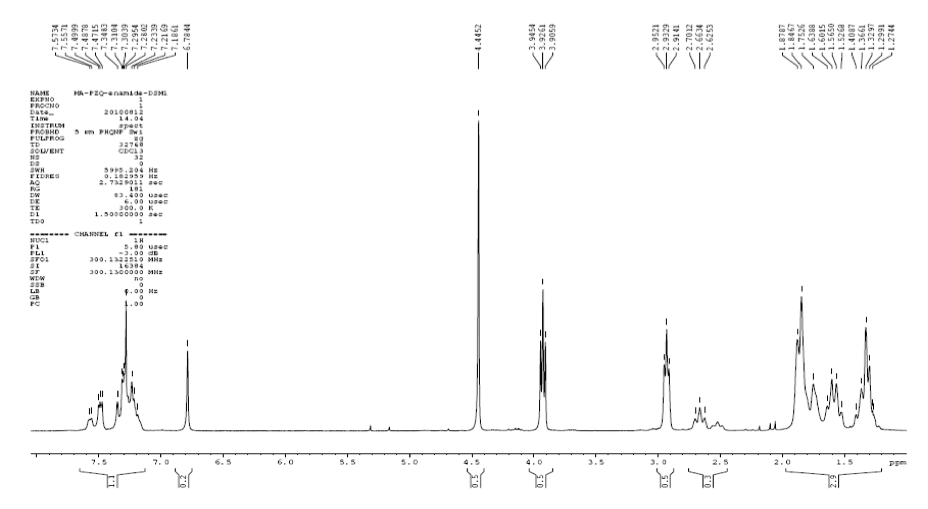

Supplement: Figure S2 — 1H NMR spectrum of 2-(cyclohexanecarbonyl)-6,7-dihydro-2H-pyrazino[2,1-a]isoquinolin-4(3H)-one, (5). (TIFF) [file pone.0047224.s002.tif]

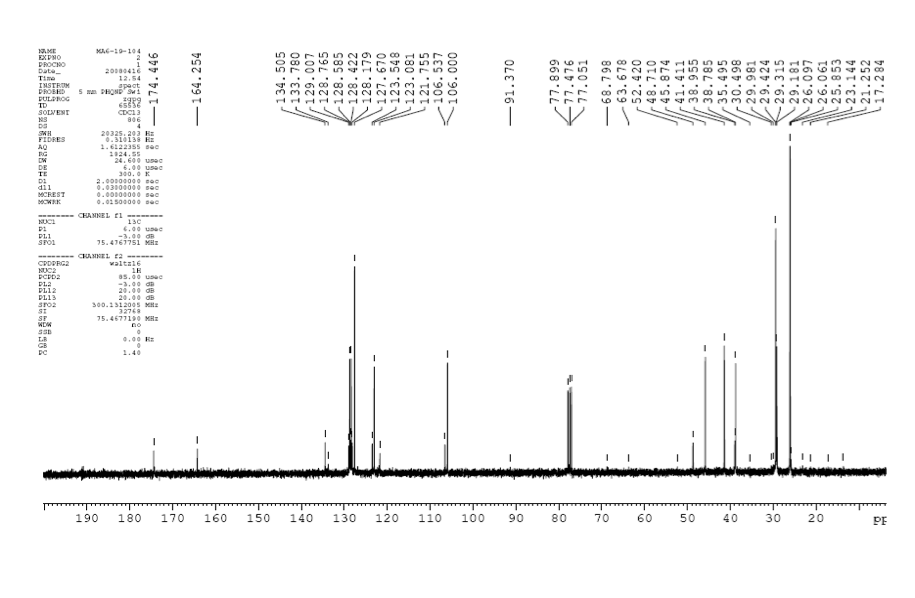

Supplement: Figure S3 — 13C NMR spectrum of 2-(cyclohexanecarbonyl)-6,7-dihydro-2H-pyrazino[2,1-a]isoquinolin-4(3H)-one, (5). (TIFF) [file pone.0047224.s003.tif]

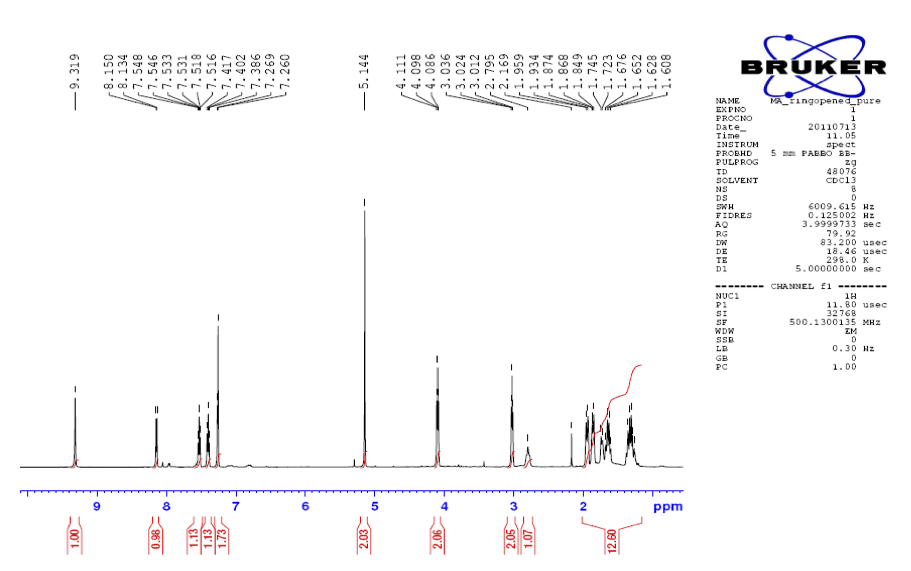

Supplement: Figure S4 — 1H NMR Spectrum of N-formyl-N-(2-oxo-2-(1-oxo-3,4-dihydroisoquinoline-2(1yl)ethyl)cyclohexanecarboxamide, (6). (TIFF) [file pone.0047224.s004.tif]

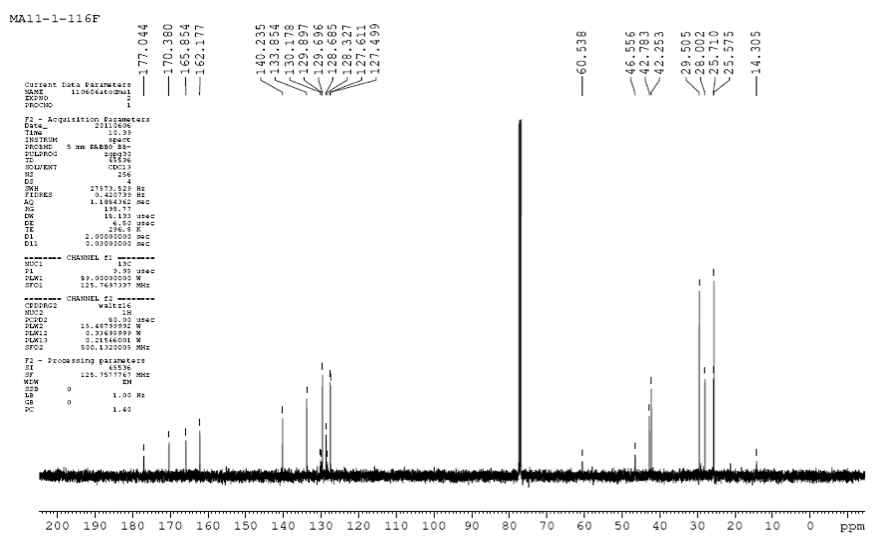

Supplement: Figure S5 — 13C NMR Spectrum of N-formyl-N-(2-oxo-2-(1-oxo-3,4-dihydroisoquinoline-2(1yl)ethyl)cyclohexanecarboxamide, (6). (TIFF) [file pone.0047224.s005.tif]
