# Supplementary material for: The Outcome of the Oxidations of Unusual Enediamide Motifs Is Governed by the Stabilities of the Intermediate Iminium Ions
Source: PLoS One. 2012 Oct 19;7(10):e47224. doi: 10.1371/journal.pone.0047224 (PMC3477162; doi:10.1371/journal.pone.0047224)
Supplement: Table S1 — B3-LYP/6-31G(d)-optimized structures and M06-2X/6-311+G(3df,2p) electronic energies for various species. (DOC) [file pone.0047224.s006.doc]

**Table S1**. B3-LYP/6-31G(d)-optimized structures and M06-2X/6-311+G(3df,2p) electronic energies for various species.

**ArCO_2_H** [E(M06-2X) = -420.8008587]

C 0.000000 0.221657 0.000000

C -1.138565 -0.596693 0.000000

C -0.995448 -1.983025 0.000000

C 0.278691 -2.556314 0.000000

C 1.414418 -1.742084 0.000000

C 1.276423 -0.356346 0.000000

H -2.124446 -0.145079 0.000000

H -1.877741 -2.617081 0.000000

H 0.386258 -3.637827 0.000000

H 2.405236 -2.187683 0.000000

H 2.143533 0.295823 0.000000

C -0.091147 1.705966 0.000000

O 0.859062 2.463136 0.000000

O -1.371163 2.161690 0.000000

H -1.302255 3.134273 0.000000

**ArCO_2_^–^** [E(M06-2X) = -420.248687]

C 0.000000 0.280089 0.000000

C -1.202260 -0.437407 0.000000

C -1.207041 -1.833928 0.000000

C 0.000973 -2.539264 0.000000

C 1.208428 -1.833183 0.000000

C 1.202702 -0.436630 0.000000

H -2.120669 0.143601 0.000000

H -2.151622 -2.378532 0.000000

H 0.001279 -3.628849 0.000000

H 2.153441 -2.377074 0.000000

H 2.120849 0.144850 0.000000

C -0.000734 1.836355 0.000000

O 1.137910 2.367999 0.000000

O -1.139871 2.366977 0.000000

**7** [E(M06-2X) = -1072.3843375]

C -3.923600 0.122762 0.688662

C -3.024411 -0.503510 -0.191310

C -3.351826 -1.741247 -0.760461

C -4.548850 -2.372750 -0.435239

C -5.435217 -1.766195 0.457838

C -5.123652 -0.523214 1.004675

H -2.671225 -2.192252 -1.475267

H -4.792569 -3.331818 -0.883368

H -6.372720 -2.252172 0.713834

H -5.823788 -0.035387 1.679088

C -3.592110 1.495351 1.227248

H -4.511620 2.047390 1.453390

H -3.024488 1.421509 2.165641

C -1.699882 0.121393 -0.512426

N -1.561756 1.501633 -0.139884

C -2.765593 2.269720 0.202874

H -3.357267 2.453404 -0.703557

H -2.421573 3.229441 0.587242

C -0.352833 2.156164 -0.206050

O -0.227573 3.351179 0.023280

C 0.867219 1.340912 -0.635959

H 0.983593 1.470197 -1.720555

H 1.720114 1.806870 -0.149448

N 0.780330 -0.075665 -0.289979

C -0.471116 -0.685232 -0.584241

H -0.472923 -1.748489 -0.371589

C 1.833856 -0.897162 0.111749

O 1.655918 -2.098650 0.270479

C 3.207322 -0.261357 0.330043

C 4.035520 -1.097967 1.322957

C 3.951407 -0.131976 -1.024115

H 3.090549 0.745184 0.753243

C 5.444488 -0.515931 1.507012

H 4.095679 -2.125993 0.948397

H 3.513787 -1.152151 2.285983

C 5.364369 0.441712 -0.837832

H 4.013193 -1.130946 -1.477278

H 3.381067 0.493898 -1.721999

C 6.182595 -0.382054 0.166749

H 6.018202 -1.149560 2.194852

H 5.375084 0.474118 1.982328

H 5.875940 0.482560 -1.807691

H 5.288537 1.479618 -0.480842

H 7.168192 0.075978 0.317687

H 6.362178 -1.384233 -0.249255

O -1.122605 -0.261961 -1.796865

**8a** [E(M06-2X) = -1072.7631843]

C -4.066278 0.212097 0.604786

C -3.002317 -0.493430 -0.025631

C -3.186710 -1.843037 -0.420884

C -4.403449 -2.467748 -0.203739

C -5.441922 -1.770497 0.427619

C -5.270154 -0.442869 0.832341

H -2.392948 -2.364908 -0.940853

H -4.551041 -3.493329 -0.525154

H -6.393534 -2.263648 0.603063

H -6.082342 0.082510 1.326219

C -3.807807 1.623023 1.065108

H -4.738208 2.196938 1.111411

H -3.393029 1.613861 2.083447

C -1.732243 0.161205 -0.199020

N -1.634696 1.482817 -0.103604

C -2.849751 2.320142 0.114167

H -3.304006 2.481256 -0.870629

H -2.507515 3.281553 0.492180

C -0.397816 2.215022 -0.358306

O -0.417671 3.415515 -0.329069

C 0.809206 1.386858 -0.715751

H 0.865206 1.354433 -1.816403

H 1.683729 1.927355 -0.355471

N 0.730599 0.054928 -0.135452

C -0.479001 -0.673812 -0.451962

H -0.508645 -1.523657 0.234540

C 1.865984 -0.779542 -0.018893

O 1.709432 -1.985896 -0.162340

C 3.209864 -0.156332 0.302174

C 3.933292 -0.989923 1.385950

C 4.069917 -0.075105 -0.988450

H 3.067069 0.859354 0.693965

C 5.333155 -0.428046 1.674290

H 4.004687 -2.026035 1.036025

H 3.331109 -1.006451 2.302789

C 5.471416 0.478591 -0.689127

H 4.149096 -1.085860 -1.408988

H 3.573707 0.545546 -1.747445

C 6.182488 -0.338613 0.398619

H 5.829311 -1.059773 2.420021

H 5.242197 0.571215 2.124696

H 6.061642 0.484936 -1.612877

H 5.387361 1.526398 -0.365561

H 7.158356 0.106419 0.623942

H 6.379412 -1.351884 0.020742

O -0.550676 -1.107717 -1.795233

H 0.111125 -1.823561 -1.862872

**8b** [E(M06-2X) = -1072.7425063]

C 3.928540 -0.013733 -0.519986

C 2.897541 -0.577022 0.252727

C 3.016090 -1.891394 0.738529

C 4.142025 -2.653991 0.446946

C 5.161814 -2.104483 -0.334533

C 5.053499 -0.797646 -0.803560

H 2.229217 -2.321211 1.354767

H 4.223758 -3.667567 0.826323

H 6.045126 -2.691118 -0.567956

H 5.858804 -0.367111 -1.392764

C 3.834018 1.416083 -1.002281

H 4.831293 1.866715 -1.042058

H 3.431204 1.449674 -2.023863

C 1.634006 0.202795 0.599912

N 1.644555 1.544364 0.061878

C 2.940569 2.233073 -0.077967

H 3.395445 2.348121 0.913549

H 2.728777 3.226437 -0.471735

C 0.488106 2.277645 -0.046353

O 0.440987 3.470520 -0.268486

C -0.835978 1.516989 0.090329

H -1.258043 1.709108 1.082210

H -1.509567 1.928553 -0.660153

N -0.724199 0.057441 -0.096800

C 0.402507 -0.530805 0.106801

H 0.425192 -1.608139 -0.032405

C -1.926005 -0.851518 -0.501624

O -1.635034 -1.971820 -0.791714

C -3.289842 -0.239817 -0.397035

C -4.288062 -0.971529 -1.321216

C -3.757435 -0.311400 1.093527

H -3.247048 0.817263 -0.683669

C -5.698869 -0.385921 -1.161609

H -4.289621 -2.037556 -1.065561

H -3.953056 -0.895645 -2.361982

C -5.174054 0.269195 1.232569

H -3.749786 -1.363477 1.405860

H -3.061065 0.224330 1.750998

C -6.171807 -0.429324 0.298505

H -6.390549 -0.941904 -1.803788

H -5.707302 0.652620 -1.522399

H -5.491069 0.173026 2.277211

H -5.146987 1.345347 1.010246

H -7.157411 0.039862 0.389944

H -6.292413 -1.475605 0.612456

O 1.360897 0.190599 2.010681

H 2.167834 -0.081928 2.483272

**10** [E(M06-2X) = -1109.2592563]

C 1.851063 1.332341 0.754021

C 1.002647 0.230642 0.112526

C -0.327640 1.991742 -1.023135

C 0.741453 2.940608 -0.724958

H 0.782522 -0.483422 0.905199

H -0.811743 2.010534 -1.997992

H 1.060431 3.664625 -1.474000

N 1.758420 2.599792 0.197872

N -0.290983 0.729010 -0.378522

C 2.510613 3.697382 0.804905

H 1.892536 4.253072 1.519958

H 3.367732 3.273866 1.326770

H 2.852016 4.381333 0.021135

C 1.780248 -0.511300 -1.015108

H 1.050087 -1.173461 -1.493748

H 2.102942 0.217091 -1.769519

O 2.624800 1.077199 1.660451

O -0.594938 3.167186 -0.227708

C -1.402831 -0.097522 -0.267388

O -1.301670 -1.228213 0.180865

C -2.729068 0.502434 -0.757303

H -2.784390 1.555004 -0.460658

H -2.699397 0.495254 -1.857897

C -3.951368 -0.241012 -0.267946

C -4.227089 -1.544166 -0.702259

C -4.839357 0.374220 0.620891

C -5.365775 -2.213852 -0.257925

H -3.540188 -2.038775 -1.382815

C -5.981323 -0.293799 1.067426

H -4.636746 1.385443 0.966956

C -6.247578 -1.590412 0.628293

H -5.564760 -3.225075 -0.602979

H -6.660527 0.200681 1.756880

H -7.135952 -2.112938 0.973025

C 2.958897 -1.324114 -0.526984

C 4.274933 -0.892013 -0.723071

C 2.741556 -2.540784 0.135126

C 5.352245 -1.654294 -0.268255

H 4.459140 0.049063 -1.236880

C 3.814769 -3.302913 0.593674

H 1.722263 -2.888877 0.289769

C 5.124794 -2.861620 0.392458

H 6.368221 -1.303644 -0.430159

H 3.628931 -4.243721 1.105158

H 5.962140 -3.456445 0.747552

**11a** [E(M06-2X) = -1109.6072405]

C -2.285355 1.242541 -0.504732

C -1.076442 0.330595 -0.294550

C 0.127085 2.282129 0.503194

C -1.096713 3.158848 0.484389

H -0.873538 -0.197771 -1.225133

H 1.053826 2.764159 0.794450

H -1.193794 3.677281 1.447506

N -2.298437 2.446630 0.186772

N 0.163576 1.078562 0.051616

C -3.522826 3.258755 0.109785

H -3.550811 3.851099 -0.809239

H -4.381450 2.589432 0.126319

H -3.556994 3.922558 0.978900

C -1.362073 -0.712234 0.845340

H -0.407003 -1.180811 1.103824

H -1.709399 -0.162136 1.727547

O -3.239536 0.827242 -1.125440

O -0.742390 4.085996 -0.545566

H -0.746122 4.993685 -0.198043

C 1.491786 0.320604 -0.180577

O 1.386286 -0.711023 -0.763193

C 2.749422 0.983401 0.344613

H 2.887080 1.927200 -0.202326

H 2.589507 1.249168 1.398143

C 3.970332 0.099712 0.194927

C 4.362748 -0.736701 1.245371

C 4.709527 0.112972 -0.992797

C 5.484421 -1.554340 1.108628

H 3.798302 -0.747420 2.175368

C 5.830241 -0.705539 -1.128821

H 4.415016 0.764479 -1.812493

C 6.218168 -1.540159 -0.078847

H 5.786524 -2.197149 1.929930

H 6.401884 -0.687410 -2.051770

H 7.093444 -2.174132 -0.184063

C -2.355733 -1.778584 0.445143

C -3.681252 -1.728246 0.890554

C -1.946956 -2.844929 -0.367900

C -4.585570 -2.727531 0.530666

H -4.008448 -0.909218 1.526685

C -2.851418 -3.840133 -0.732938

H -0.915419 -2.901507 -0.709977

C -4.173124 -3.782781 -0.283624

H -5.610443 -2.681265 0.886831

H -2.523640 -4.663835 -1.360267

H -4.876991 -4.560741 -0.564245

**11b** [E(M06-2X) = -1109.6049788]

C -2.156708 1.296321 -0.520558

C -0.999515 0.363054 -0.318086

C 0.286183 2.487699 -0.016237

C -0.964877 3.075683 0.591248

H -0.868809 -0.209029 -1.239145

H 1.155630 2.851190 0.532567

H -0.921825 4.005865 1.153543

N -2.109733 2.549408 0.331835

N 0.254248 1.057462 0.028478

C -3.407351 3.155893 0.688005

H -3.231954 4.063004 1.266380

H -3.942532 3.381036 -0.236036

H -3.985296 2.432237 1.266067

C -1.424318 -0.640973 0.829290

H -0.515388 -1.206337 1.046590

H -1.676669 -0.064849 1.727845

O -3.158352 1.101109 -1.136753

C 1.435283 0.278363 -0.115118

O 1.332840 -0.887175 -0.430235

C 2.771834 0.973493 0.166671

H 2.919364 1.761515 -0.586996

H 2.709495 1.480185 1.139091

C 3.947616 0.021977 0.154339

C 4.439685 -0.503625 1.353445

C 4.553974 -0.343937 -1.052360

C 5.520852 -1.385421 1.347626

H 3.979734 -0.222142 2.298436

C 5.634115 -1.225317 -1.060239

H 4.181455 0.059251 -1.991483

C 6.119392 -1.747915 0.140332

H 5.896797 -1.784462 2.285202

H 6.098522 -1.501113 -2.002477

H 6.963357 -2.431417 0.134663

C -2.544867 -1.571224 0.439520

C -3.835106 -1.410445 0.960146

C -2.298026 -2.626029 -0.452579

C -4.862601 -2.282456 0.597648

H -4.035487 -0.612628 1.673618

C -3.324085 -3.494011 -0.819494

H -1.295090 -2.773391 -0.847657

C -4.608838 -3.323093 -0.296007

H -5.856230 -2.152322 1.016394

H -3.120390 -4.310227 -1.506344

H -5.406200 -4.003953 -0.578871

O 0.217144 3.055508 -1.323282

H 1.111015 3.245748 -1.656962

**12a** [E(M06-2X) = -1530.097563]

C -1.631396 -1.918431 0.005003

C -1.638378 -0.382337 -0.030928

C 0.471047 -0.656301 -1.339573

C -0.308666 -1.769124 -2.062673

H -1.648835 -0.051156 1.007732

H -1.035424 -1.263471 -2.716906

N -1.021469 -2.554774 -1.044183

N -0.442052 0.211973 -0.679926

C -1.129305 -4.002866 -1.190119

H -0.149341 -4.482892 -1.118021

H -1.771201 -4.354592 -0.383044

H -1.568466 -4.261544 -2.159012

C -2.944061 0.126208 -0.720408

H -2.774248 1.176653 -0.972545

H -3.093467 -0.415412 -1.662756

O -2.226542 -2.500602 0.902724

O 0.476512 -2.569106 -2.883973

H 1.414650 -2.502471 -2.603271

C -0.118946 1.516938 -0.308949

O -0.853781 2.147811 0.432938

C 1.178166 2.109666 -0.883271

H 2.019442 1.517116 -0.498668

H 1.184736 1.979115 -1.972146

C 1.369038 3.571520 -0.548434

C 1.256959 4.546787 -1.544387

C 1.678060 3.974591 0.756889

C 1.450222 5.897430 -1.247687

H 1.017559 4.248973 -2.563083

C 1.871617 5.321564 1.057341

H 1.760012 3.228921 1.543389

C 1.758973 6.288088 0.054924

H 1.360546 6.641088 -2.035077

H 2.109869 5.617867 2.075437

H 1.911502 7.338211 0.289205

C -4.175923 0.015827 0.150950

C -5.131942 -0.984893 -0.052474

C -4.378385 0.941787 1.184520

C -6.266696 -1.062318 0.756501

H -4.986847 -1.711631 -0.848453

C -5.509429 0.865038 1.995998

H -3.640555 1.724405 1.347087

C -6.458542 -0.137586 1.783425

H -6.999456 -1.846435 0.584131

H -5.652948 1.591615 2.791717

H -7.342279 -0.196319 2.413436

H 1.095645 -0.129364 -2.060311

O 1.364633 -1.199069 -0.294156

C 2.578738 -1.674059 -0.629857

O 2.901081 -1.921777 -1.787252

C 3.475904 -1.861657 0.535370

C 3.070588 -1.557919 1.844158

C 4.765246 -2.361874 0.298119

C 3.953194 -1.753643 2.903781

H 2.071107 -1.178448 2.023759

C 5.643367 -2.551371 1.360978

H 5.058209 -2.593124 -0.720500

C 5.238014 -2.247676 2.663770

H 3.638762 -1.521503 3.917105

H 6.641962 -2.936581 1.176246

H 5.923716 -2.397817 3.493273

**12b** [E(M06-2X) = -1530.0900757]

C 0.094249 1.991051 -0.587512

C -1.135374 1.148228 -0.229775

C 0.510166 -0.712512 -0.191899

C 1.453390 0.287197 0.515542

H -1.806090 1.183978 -1.090122

H 0.673254 -1.683007 0.291803

N 1.322219 1.594413 -0.078635

N -0.838953 -0.266861 0.089463

C 2.403703 2.571156 0.016160

H 2.805100 2.600017 1.035403

H 3.214445 2.345384 -0.683886

H 1.982111 3.541164 -0.245933

C -1.843923 1.836024 0.985752

H -2.469229 1.070005 1.449889

H -1.086816 2.129212 1.724356

O -0.050025 3.054395 -1.167929

C -1.917037 -1.142308 -0.014671

O -3.054556 -0.718643 -0.153141

C -1.607459 -2.646389 0.063736

H -0.982329 -2.897397 -0.803894

H -0.996238 -2.844693 0.952218

C -2.842950 -3.516738 0.093836

C -3.165408 -4.251629 1.239229

C -3.674292 -3.623146 -1.028913

C -4.291215 -5.077451 1.267695

H -2.529233 -4.179148 2.119123

C -4.798932 -4.445641 -1.004682

H -3.441932 -3.051108 -1.922948

C -5.111131 -5.176917 0.144212

H -4.524371 -5.641834 2.166882

H -5.433897 -4.515733 -1.883985

H -5.987578 -5.819339 0.161722

C -2.707064 3.021109 0.613656

C -2.312602 4.334484 0.889593

C -3.946262 2.805994 -0.007611

C -3.133721 5.412275 0.554247

H -1.352030 4.516329 1.365797

C -4.766365 3.881078 -0.347496

H -4.260643 1.786999 -0.220209

C -4.363212 5.188669 -0.066069

H -2.810709 6.426298 0.775978

H -5.724317 3.697559 -0.827553

H -5.003878 6.026888 -0.327711

O 0.658747 -0.791659 -1.583705

H 1.596978 -0.626229 -1.819128

H 1.186570 0.286461 1.580409

O 2.835387 -0.142758 0.599256

C 3.662637 -0.315184 -0.460066

O 3.306348 -0.288857 -1.627381

C 5.067355 -0.547106 -0.038584

C 5.463618 -0.521047 1.307222

C 6.015343 -0.797478 -1.042503

C 6.797610 -0.743688 1.640637

H 4.729305 -0.327171 2.080530

C 7.346728 -1.018694 -0.702922

H 5.690329 -0.813147 -2.077324

C 7.738802 -0.992094 0.638285

H 7.103999 -0.722823 2.682490

H 8.078707 -1.211840 -1.481698

H 8.778500 -1.164964 0.902593

**13a** [E(M06-2X) = -1493.2130942]

C -0.940189 2.921757 -0.906836

C -0.942219 2.037712 0.182626

C -1.194439 2.527277 1.473028

C -1.402273 3.884858 1.692018

C -1.363684 4.773543 0.614516

C -1.141765 4.288519 -0.670387

H -1.234281 1.834523 2.308964

H -1.590973 4.247817 2.698214

H -1.519364 5.836900 0.775023

H -1.135302 4.973647 -1.515320

C -0.761258 2.403617 -2.317224

H -1.369809 2.993120 -3.013531

H 0.284164 2.512641 -2.638663

C -0.713362 0.530234 0.008797

N -0.410865 0.197481 -1.361850

C -1.156161 0.932998 -2.391511

H -2.232239 0.810864 -2.220120

H -0.903764 0.480536 -3.349514

C 0.432866 -0.821923 -1.768007

O 0.502655 -1.200639 -2.926477

C 1.332653 -1.401928 -0.688575

H 0.860726 -2.305471 -0.283937

H 2.254196 -1.705225 -1.178044

N 1.571758 -0.422440 0.356595

C 0.380751 0.026971 1.057157

H 0.702118 0.884189 1.640097

C 2.805375 0.053627 0.786790

O 2.877724 0.861281 1.705597

C 4.071263 -0.464211 0.097559

C 5.200695 0.577109 0.208838

C 4.510999 -1.810119 0.727318

H 3.877840 -0.631553 -0.970191

C 6.502487 0.061823 -0.420906

H 5.357010 0.810414 1.267884

H 4.888318 1.513705 -0.268593

C 5.819962 -2.321894 0.106485

H 4.646604 -1.655257 1.806474

H 3.721021 -2.564046 0.617679

C 6.939283 -1.274533 0.197522

H 7.293907 0.812310 -0.300402

H 6.361698 -0.069146 -1.504515

H 6.124212 -3.252140 0.602801

H 5.644409 -2.574896 -0.949797

H 7.846297 -1.646329 -0.295754

H 7.198917 -1.115323 1.254326

O -0.107422 -0.895395 1.989941

H -0.464237 -1.660706 1.500693

O -2.073100 0.001347 0.343560

C -2.403064 -1.294077 0.212832

O -1.590479 -2.202442 0.078957

C -3.872803 -1.515595 0.260147

C -4.783390 -0.457531 0.400089

C -4.341775 -2.833531 0.159705

C -6.150815 -0.721442 0.438338

H -4.414469 0.558699 0.480930

C -5.709280 -3.090947 0.196511

H -3.621400 -3.637455 0.052005

C -6.614652 -2.035455 0.336007

H -6.855615 0.097939 0.547894

H -6.070357 -4.112366 0.117100

H -7.682145 -2.237042 0.365445

**13b** [E(M06-2X) = -1493.2172121]

C 3.582778 1.631616 -0.272152

C 2.439880 1.455542 0.527396

C 2.526445 1.673876 1.906815

C 3.734065 2.055881 2.487903

C 4.870848 2.220505 1.696857

C 4.788409 2.013720 0.321215

H 1.657979 1.546976 2.538673

H 3.782831 2.222966 3.560003

H 5.813933 2.518885 2.146808

H 5.664446 2.159120 -0.306566

C 3.445679 1.467589 -1.764807

H 4.276293 1.956736 -2.284977

H 3.462312 0.406536 -2.049170

C 1.159992 0.956209 -0.139509

N 1.004975 1.475364 -1.483266

C 2.126163 2.092709 -2.206942

H 2.136033 3.171698 -2.008337

H 1.932596 1.946250 -3.270083

C -0.159061 1.249592 -2.176971

O -0.318219 1.581859 -3.342967

C -1.262979 0.478813 -1.454885

H -2.193012 0.804590 -1.920188

H -1.118447 -0.584070 -1.693016

N -1.295015 0.694527 -0.008458

C -0.119989 1.251011 0.668494

H -0.061090 0.750816 1.633784

C -2.457315 0.758530 0.725946

O -2.455217 1.296087 1.838108

C -3.733225 0.144670 0.158983

C -4.391073 -0.757990 1.226377

C -4.706415 1.266025 -0.279067

H -3.499888 -0.478065 -0.713419

C -5.731177 -1.324246 0.735492

H -4.539005 -0.163227 2.135150

H -3.707443 -1.574106 1.492692

C -6.048810 0.696561 -0.761058

H -4.867198 1.930074 0.580140

H -4.251081 1.879041 -1.068640

C -6.692287 -0.208986 0.299287

H -6.186947 -1.931367 1.527740

H -5.554160 -2.002521 -0.112755

H -6.725286 1.519576 -1.023667

H -5.888619 0.118981 -1.683700

H -7.625240 -0.639458 -0.085966

H -6.964250 0.397366 1.175604

O -0.207407 2.647240 0.821731

H -0.946513 2.761036 1.449247

O 1.271816 -0.517148 -0.355880

C 1.365938 -1.403432 0.668790

O 1.282567 -1.113130 1.845540

C 1.570621 -2.796571 0.172101

C 1.678162 -3.107354 -1.191003

C 1.652348 -3.819593 1.127406

C 1.864143 -4.429784 -1.590365

H 1.616912 -2.314986 -1.928437

C 1.839008 -5.139075 0.724094

H 1.567835 -3.558371 2.177019

C 1.944492 -5.445938 -0.635208

H 1.947185 -4.667664 -2.647217

H 1.902167 -5.928586 1.467742

H 2.089805 -6.476011 -0.949770

**13c** [E(M06-2X) = -1493.2182725]

C -3.561134 -1.896458 -0.704468

C -2.917900 -0.929598 0.087104

C -3.546019 0.304368 0.300747

C -4.769898 0.599289 -0.292739

C -5.393766 -0.349051 -1.104002

C -4.793972 -1.590913 -1.292816

H -3.082634 1.045073 0.943108

H -5.235422 1.564922 -0.117404

H -6.350744 -0.129537 -1.569425

H -5.291960 -2.349028 -1.893038

C -2.959925 -3.276420 -0.829056

H -3.746416 -4.015435 -1.019774

H -2.254762 -3.332196 -1.670324

C -1.539010 -1.181484 0.715546

N -1.197873 -2.598209 0.700016

C -2.224911 -3.614231 0.463465

H -2.927588 -3.650470 1.306439

H -1.706335 -4.571899 0.412865

C 0.030670 -2.994479 1.175853

O 0.318392 -4.152094 1.440506

C 1.042940 -1.860862 1.296753

H 0.905410 -1.350803 2.256951

H 2.028356 -2.316660 1.285033

N 0.888396 -0.932015 0.177558

C -0.430640 -0.397653 -0.072492

H -0.636674 -0.394244 -1.141273

C 1.896601 -0.512179 -0.683318

O 1.637094 0.288119 -1.574069

C 3.320488 -1.029130 -0.467802

C 4.130123 -0.931871 -1.774260

C 4.013396 -0.222315 0.659543

H 3.295304 -2.085474 -0.169157

C 5.582080 -1.387610 -1.567677

H 4.103859 0.105381 -2.126770

H 3.646692 -1.532360 -2.553924

C 5.469161 -0.670166 0.860714

H 3.987246 0.841447 0.385308

H 3.458037 -0.316522 1.601381

C 6.271307 -0.595881 -0.446534

H 6.139375 -1.279170 -2.506518

H 5.599977 -2.459220 -1.318423

H 5.941212 -0.052969 1.635710

H 5.480019 -1.704260 1.236038

H 7.290853 -0.968554 -0.286243

H 6.365854 0.456302 -0.752470

O -1.524656 -0.752646 2.081187

H -1.348206 0.204059 2.068519

O -0.463728 0.979781 0.383853

C -0.744398 1.957457 -0.545451

O -1.217408 1.725588 -1.632365

C -0.439486 3.317690 -0.023813

C 0.213409 3.532941 1.198112

C -0.824510 4.413291 -0.809838

C 0.473297 4.833004 1.628287

H 0.523225 2.684894 1.798193

C -0.564676 5.710058 -0.375475

H -1.321380 4.224911 -1.755726

C 0.083558 5.921525 0.844495

H 0.982550 4.997210 2.573868

H -0.865018 6.556259 -0.987000

H 0.287255 6.933998 1.182890

**13d** [E(M06-2X) = -1493.2225511]

C 2.976840 -2.082670 0.147237

C 1.767316 -2.068864 -0.566476

C 1.769313 -2.335771 -1.943308

C 2.959023 -2.605301 -2.613059

C 4.164125 -2.613656 -1.907623

C 4.165843 -2.359738 -0.538235

H 0.829392 -2.330253 -2.486223

H 2.945371 -2.803933 -3.680760

H 5.098003 -2.825049 -2.421419

H 5.100784 -2.382331 0.017118

C 2.966141 -1.844782 1.638638

H 3.817943 -2.347478 2.111252

H 3.059406 -0.772650 1.859551

C 0.427225 -1.749552 0.093630

N 0.528481 -1.724911 1.553757

C 1.659329 -2.362543 2.231676

H 1.598375 -3.454209 2.118052

H 1.559747 -2.127136 3.291177

C -0.447072 -1.131661 2.310643

O -0.445853 -1.132562 3.535587

C -1.621239 -0.466579 1.589572

H -2.463440 -1.159418 1.700205

H -1.848751 0.426739 2.179312

N -1.411488 -0.141904 0.179226

C -0.113567 -0.377725 -0.376582

C -2.413816 0.325864 -0.664823

O -2.185682 0.569764 -1.842257

C -3.819743 0.482732 -0.087457

C -4.497059 1.743725 -0.660069

C -4.646891 -0.782417 -0.430766

H -3.782455 0.581285 1.003993

C -5.950891 1.868042 -0.182126

H -4.458461 1.689224 -1.753830

H -3.924298 2.634678 -0.372910

C -6.101527 -0.652585 0.043925

H -4.619203 -0.919618 -1.519877

H -4.179301 -1.674043 0.007476

C -6.767975 0.611662 -0.518310

H -6.413674 2.754410 -0.634102

H -5.968085 2.030384 0.906253

H -6.666951 -1.546113 -0.249969

H -6.124203 -0.617218 1.143422

H -7.790186 0.706067 -0.130419

H -6.853310 0.518225 -1.610802

O -0.577864 -2.668882 -0.336003

H -0.202807 -3.562003 -0.264923

H -0.185969 -0.332111 -1.459841

O 0.800526 0.642420 0.085779

C 1.490924 1.354475 -0.853624

O 1.452162 1.118164 -2.041260

C 2.300425 2.442958 -0.236346

C 2.282444 2.710223 1.140077

C 3.097551 3.221576 -1.086976

C 3.058400 3.746898 1.656436

H 1.659413 2.111878 1.795206

C 3.870813 4.255902 -0.566715

H 3.093570 2.999925 -2.149048

C 3.852430 4.519510 0.805679

H 3.041421 3.953702 2.722956

H 4.486880 4.857563 -1.229202

H 4.455328 5.327610 1.211590
