# Supplementary material for: The Outcome of the Oxidations of Unusual Enediamide Motifs Is Governed by the Stabilities of the Intermediate Iminium Ions
Source: PLoS One. 2012 Oct 19;7(10):e47224. doi: 10.1371/journal.pone.0047224 (PMC3477162; doi:10.1371/journal.pone.0047224)
Supplement: Text S1 — General Experimental Information. (DOC) [file pone.0047224.s007.doc]

**Supporting Information for**

**Text S1**

***General Experimental Information:***

^1^H Nuclear Magnetic Resonance spectra were recorded on a Bruker AVANCE DPX200 (200 MHz) or a Bruker AVANCE DPX300 or AM300 (300 MHz) spectrometer at 300 K. Signals are reported in the order chemical shift (ppm downfield with respect to internal TMS), multiplicity, integration, coupling constants *J* (Hz) and assignments. (s = singlet, d = doublet, dd = doublet of doublets (etc), t = triplet, q = quartet, m = multiplet, br s = broad singlet). ^13^C Nuclear Magnetic Resonance spectra were recorded on the latter instruments (75.46 MHz). The chemical shifts are reported relative to chloroform (δ 77.0 ppm).

Flash column chromatography was performed using Merck Kieselgel 60 silica gel (SiO_2_, 0.040-0.063 mm). TLC analysis was performed on precoated 60F_254_ slides, and visualised by one of UV irradiation, I_2_ or ninhydrin staining.

Low-resolution electrospray ionization mass spectrometry (ESI-MS) was performed on a ThermoQuest Finnigan LCQ Deca ion trap mass spectrometer and high resolution mass spectrometry (HR-MS) was performed on a Bruker Fourier Transform Ion Cyclotron Resonance (FT-ICR) spectrometer in electrospray mode with a 4.7 T superconducting magnet by Dr. Keith Fisher at the Australian National University, Canberra, Australia.

Infrared measurements were done on a Perkin Elmer 1600 series FTIR spectrometer with compounds dissolved in dichloromethane and evaporated as a thin film on 0.5 cm sodium chloride plates. Absorption maxima are expressed in wavenumbers (cm^-1^)

X-ray crystallographic diffraction data were collected on a Bruker Smart 1000 CCD equipped with Mo-Kα (λ = 0.71073 Å) radiation and Oxford Instruments nitrogen gas (100-375 K) and helium gas (25- 375 K) cryostreams. Crystals were mounted on a mohair fibre in a thin film of perfluoropolyether oil and quench-cooled to 150 K.
